# Smoothened inhibition leads to decreased cell proliferation and suppressed tissue fibrosis in the development of benign prostatic hyperplasia

**DOI:** 10.1038/s41420-021-00501-4

**Published:** 2021-05-18

**Authors:** Jianmin Liu, Jing Yin, Ping Chen, Daoquan Liu, Weixiang He, Yan Li, Mingzhou Li, Xun Fu, Guang Zeng, Yuming Guo, Xinghuan Wang, Michael E. DiSanto, Xinhua Zhang

**Affiliations:** 1 Department of Urology, Zhongnan Hospital of Wuhan University, Wuhan, China; 2 Department of Rehabilitation, Zhongnan Hospital of Wuhan University, Wuhan, China; 3 Department of Surgery and Biomedical Sciences, Cooper Medical School of Rowan University, Camden, NJ USA

**Keywords:** Benign prostatic hyperplasia, Apoptosis, Cell growth, Cell proliferation

## Abstract

Benign prostatic hyperplasia (BPH) is a common disease in aging males. It has been proven that the Hedgehog (HH) is implied as an effective and fundamental regulatory growth factor signal for organogenesis, homeostasis, and regeneration. Smoothened (SMO), as the major control point of HH signals, activates aberrantly in most human solid tumors. However, the specific function of SMO and its downstream glioma-associated oncogene (GLI) family in BPH has not been well understood. Here, we first revealed that the SMO cascade was upregulated in BPH tissues and was localized in both the stromal and the epithelium compartments of human prostate tissues. Cyclopamine, as a specific SMO inhibitor, was incubated with BPH-1 and WPMY-1, and intraperitoneally injected into a BPH rat model established by castration with testosterone supplementation. SMO inhibition could induce cell apoptosis, cell cycle arrest at the G0/G1 phase, and a reduction of tissue fibrosis markers, both in vitro and in vivo. Finally, a tissue microarray, containing 104 BPH specimens, was constructed to analyze the correlations between the expression of SMO cascade and clinical parameters. The GLI2 was correlated positively with nocturia and negatively with fPSA. The GLI3 was in a positive relationship with International Prostate Symptom Score and nocturia. In conclusion, our study suggested that SMO cascade could play important roles in the development of BPH and it might be rediscovered as a promising therapeutic target for BPH.

## Introduction

Benign prostatic hyperplasia (BPH) refers to the nonmalignant growth of the prostate that occurred commonly in aging men and can lead to lower urinary tract symptoms (LUTS)^1^. Bothersome LUTS can interfere with activities of daily living, causing significant impairment of disease-specific quality of life. The presence of histological BPH at autopsy is ~50% in those aged 51–60 years and 70% in those aged 61–70 years^2^. Androgen levels and aging are two indispensable driving factors of BPH^3^. Despite the high prevalence, the causative factors of the initial event leading to the development of BPH remain unclear. Consequently, it is of great significance to identify new markers and new therapeutic targets for BPH.

It has been documented that robust Hedgehog (HH) signals occur frequently during embryonic development and infancy^4^, playing an instrumental role during diverse processes of cell differentiation and organ development (e.g., regulating cavernosa and chondrocyte differentiation^5,6^, coordinating lung specification, branching morphogenesis, and foregut mesenchymal differentiation^7^). New glands, which can only be seen in the embryonic period, are often found in the human hyperplastic prostate. It has been proposed that the occurrence of BPH is the “reawakening” of the embryonic process that prostate mesenchyme induced epithelial differentiation^8^. Several HH target genes have been identified in the mesenchyme of developing prostate, including the cytokine *Cxcl14*, the insulin-like growth factor-binding protein *Igfbp3*, and the delta/notch-like epidermal growth factor-related receptor *Dner*^9^. Indeed, HH pathway target genes have been implicated in the placement of prostate epithelial buds and in subsequent ductal branching and outgrowth^10^.

The activation of HH signaling cascade is initiated by binding HH ligands to the membrane receptor Patched1 (PTCH1), thus releasing smoothened (SMO) to be activated by phosphorylation. SMO ultimately decreases the interaction between suppressor of fused homolog and glioma-associated oncogenes (GLIs). GLI proteins belong to zinc-finger transcription factors and are the terminal effectors of SMO cascade. This family consists of three major regulators in mammals, i.e., GLI1–3. GLI1 acts only as a transcriptional activator (GLI-A), whereas GLI2 and GLI3 can be processed into both transcriptional activators and repressors (GLI-R), depending on the specific cell context and on the activation state of HH signaling^11^.

The transmembrane protein SMO, a member of the Frizzled class of G-protein-coupled receptor superfamily, is a critical control point of the HH signaling pathway. The accumulation of activated SMO suppresses the generation of GLI-R forms and allows GLI-A proteins to translocate into the nucleus and transcriptionally activate their target genes that include HH-GLI pathway autoregulation (*GLI1*, *PTCH1*, *HHIP1*), proliferation and differentiation (e.g., *cyclin D1*, *cyclin E*, *E2F1*, *FOXM1*, *MYC*, *IGFBP3*), apoptosis (e.g., *Bcl-2*), epithelial-to-mesenchymal transition (e.g., *SNAIL*, *MMP9*, *ZEB1*, *ZEB2*), and stem cell self-renewal (e.g., *NANOG*, *SOX2*)^12,13^. Contrarily, in the absence of HH ligand, PTCH1 represses the HH signals by inhibiting the activity of SMO. Therefore, SMO is the central transductor in HH signaling and is of major academic and pharmaceutical interest. In addition, the actions of HH signaling, especially SMO and its downstream GLI family, on growth, epithelial–mesenchymal interactions, and fibrosis in BPH are not well documented.

The current study concentrated on SMO and its downstream GLI family of proteins in the regulation of BPH development. Our study extended the functional activities of SMO to human prostate tissues and human prostate cell lines. Furthermore, in vivo effect of SMO cascade was investigated with SMO inhibitor CYC treating BPH rat model established by castration with testosterone supplementation.

## Results

### The expression and localization of SMO and GLI1–3 in human prostate tissues and cell lines

**Fig. 1:**
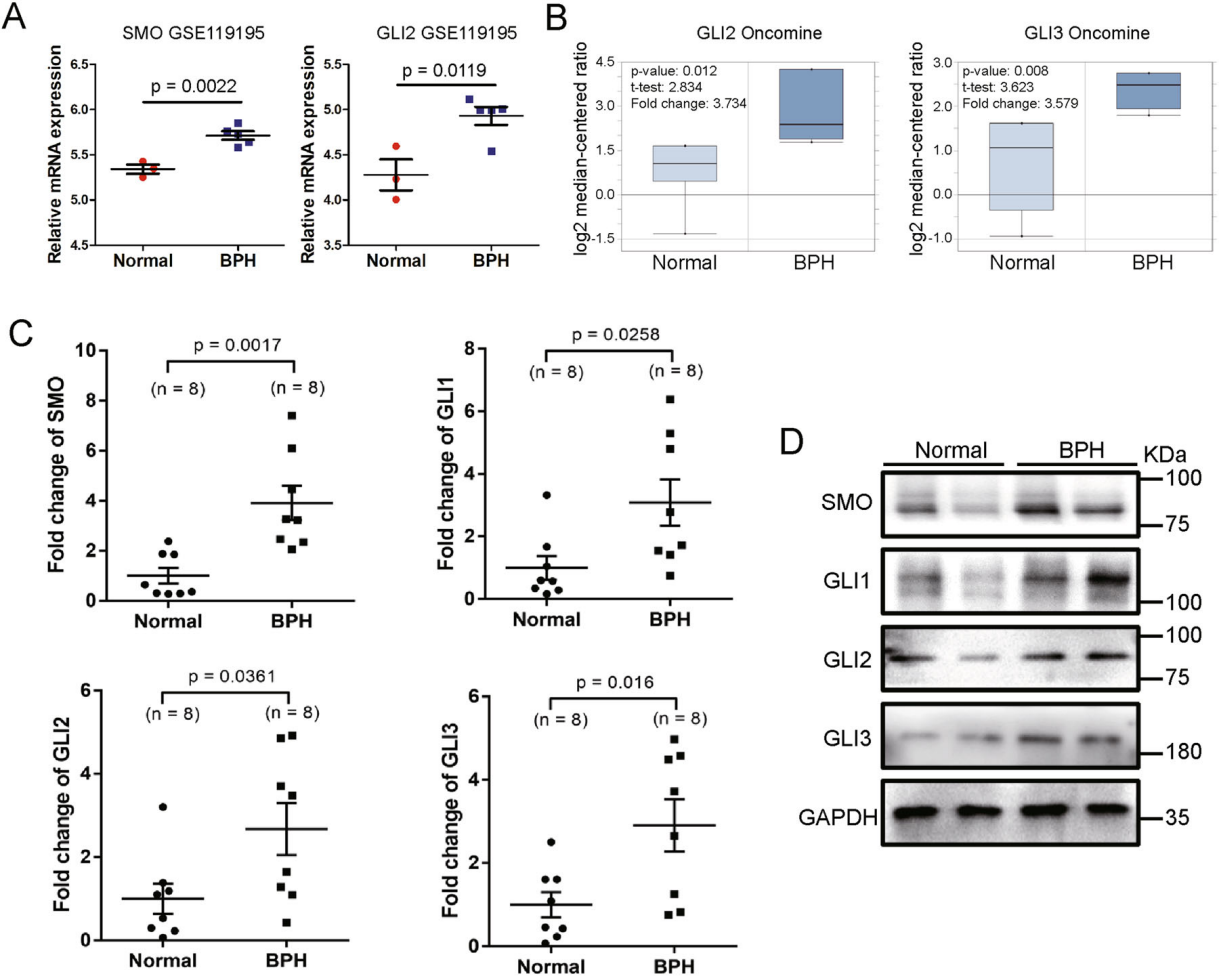
SMO and GLI1–3 are strongly upregulated in BPH tissues compared with the normal ones. **A** The mRNA expression of SMO and GLI2 in BPH and NP through GSE119195 dataset. **B** The mRNA expression of GLI2 and GLI3 in BPH and NP through Tomlins Prostate from Oncomine database. **C**, **D** The relative mRNA and protein expression of SMO, GLI1, GLI2, and GLI3 in BPH tissues versus normal prostate tissues. Two-tailed Student’s *t* test.

**Fig. 2:**
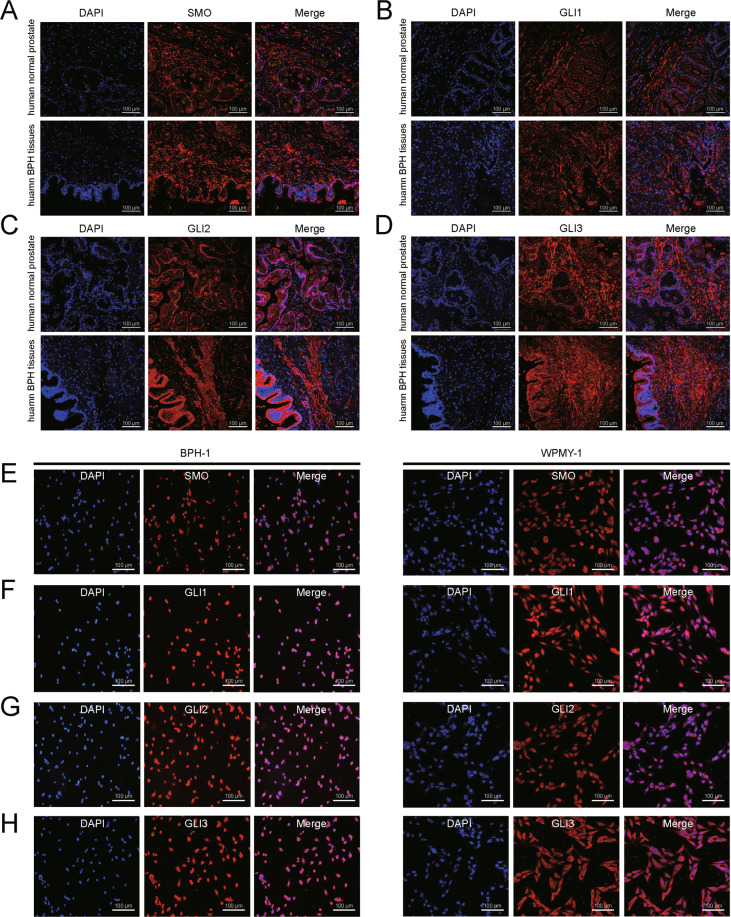
Immunofluorescence localization of SMO and GLI family in human prostate tissues and human prostate cell lines. **A**–**D** Immunolocalization of SMO, GLI1, GLI2, and GLI3 for normal human prostate (above) and BPH prostate (below). **E**–**H** Immunofluorescence of SMO, GLI1, GLI2, and GLI3 in BPH-1 and WPMY-1 cells. The scale bars are 100 μm.

Three Gene Expression Omnibus (GEO) datasets (GSE6099, GSE3868, and GSE119195) and the Oncomine database were explored to analyze the differentially expressed SMO cascade genes between BPH specimens and normal prostate (NP) specimens. As shown in Fig. 1A, the expression of SMO and GLI2 at transcriptional levels was significantly upregulated in BPH when compared with NP through GSE119195 dataset. The messenger RNA (mRNA) expression of GLI2 and GLI3 was significantly increased in BPH observed in *Tomlins Prostate*^14^ from the Oncomine database (Fig. 1B). Normal and hyperplastic prostate were harvested (*n* = 16, eight control samples and eight BPH samples) from our institute. In the hyperplastic prostate, the mRNA level of SMO and GLI1–3 was significantly increased over 2-fold and the protein level of these molecules was consistently increased (Fig. 1C, D). Meanwhile, the immunofluorescence staining showed that SMO and GLI1–3 were localized in both the stromal and the epithelium compartments of the human prostate (Fig. 2A–D). Consistently, they were expressed in epithelium cell BPH-1 and stromal cell WPMY-1 (Fig. 2E–H). Hence, epithelial and stromal cells were used in our subsequent studies.

### Human prostate cells with CYC treatment increased cell apoptosis and induced G0/G1 arrest

**Fig. 3:**
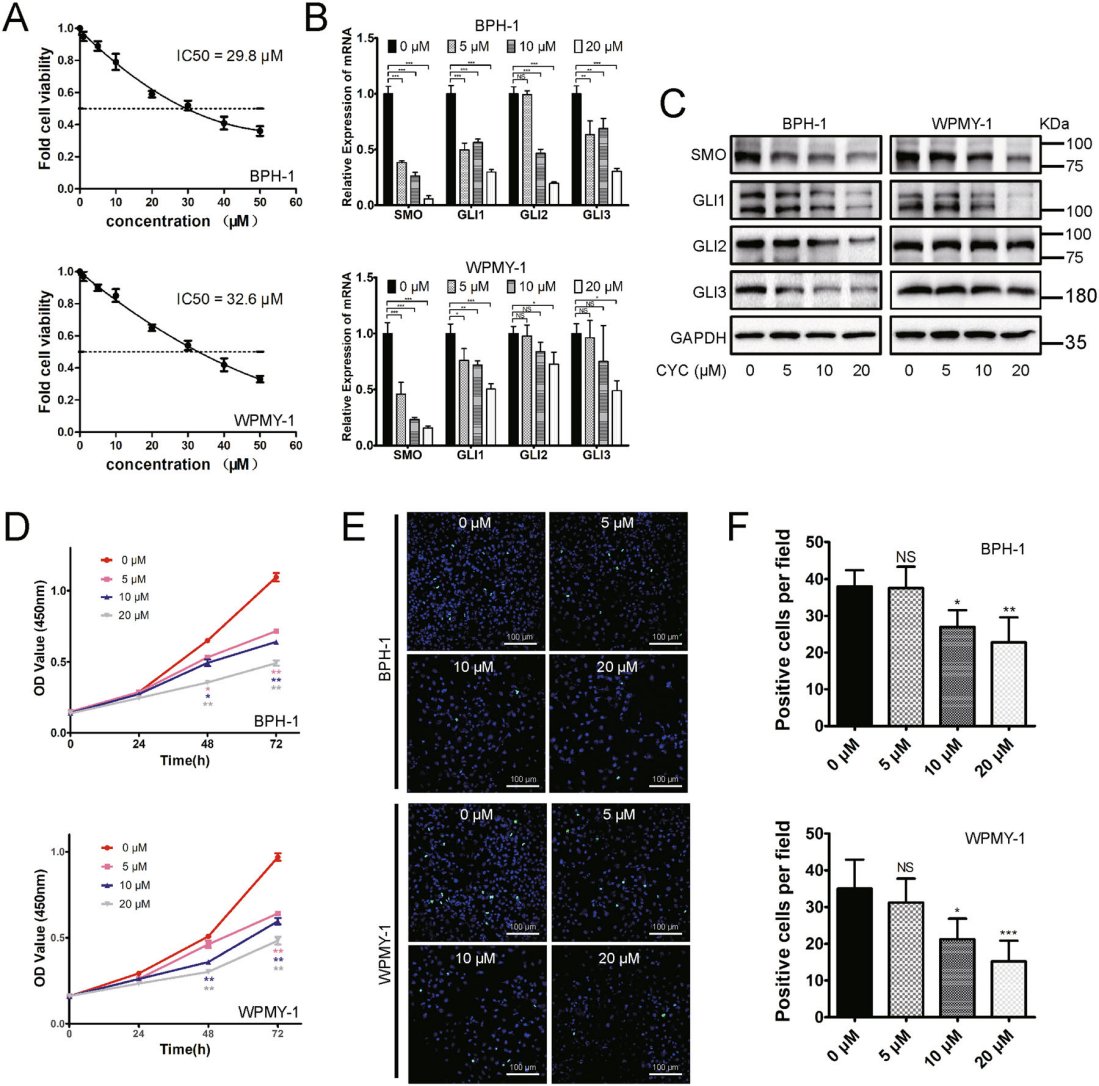
Typical dose–response curves for the inhibition of proliferation by CYC in BPH-1 and WPMY-1 cells. **A** The inhibition of proliferation by CYC from 0 to 50 μM, the IC50 for the cytotoxic effect of BPH-1 and WPMY-1 cells is 29.8 and 32.6 μM, respectively. **B**, **C** The relative mRNA and protein level of SMO, GLI1, GLI2, and GLI3 in BPH-1 and WPMY-1 after CYC treated. **D** The cell viability of BPH-1 and WPMY-1 after CYC treated at different time points by CCK-8 assay. **E** The Ki-67 staining of BPH-1 and WPMY-1 after CYC treated. **F** Statistical analysis of Ki-67-positive cells per field in BPH-1 and WPMY-1 after CYC treated. For 5, 10, and 20 μM CYC compared with 0 μM CYC, NS means no significant difference, *p < 0.05, **p < 0.01, ***p < 0.001; one-way ANOVA. The scale bars are 100 μm.

**Fig. 4:**
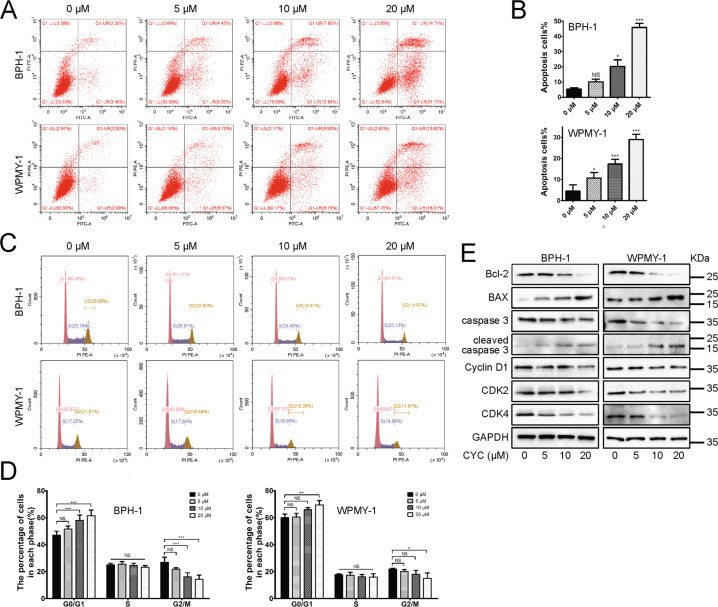
Downregulation of SMO induces cell apoptosis and cell cycle arrest. **A** Flow cytometry analysis of the cell apoptosis in BPH-1 and WPMY-1 after CYC treated for 48 h. PI PE-A in *y*-axis stands for the fluorescence intensity of propidine iodide (PI) and FITC-A in *x*-axis stands for the fluorescence intensity of fluorescein isothiocyanate (FITC)-labeled Annexin V. Calculation area of the apoptosis rate was the percentage of Annexin V+/PI+ cells. **B** Statistical analysis reveals the apoptotic rate (%) of BPH-1 and WPMY-1 after CYC treated. **C** Flow cytometry analysis of the cell cycle in BPH-1 and WPMY-1 after CYC treated for 48 h. Percentages (%) of cell populations at different stages of cell cycles were listed within the panels. **D** Statistical analysis of the percentages (%) of cell populations at different stages of the cell cycles in BPH-1 and WPMY-1 after CYC treated. **E** Immunoblot assay of apoptosis-related and cell cycle-related protein in BPH-1 and WPMY-1 after CYC treated. NS means no significant difference, *p < 0.05, **p < 0.01, ***p < 0.001; one-way ANOVA.

Human cell lines were treated with the SMO inhibitor CYC. The Cell Counting Kit-8 (CCK-8) assay was applied to detect BPH-1 and WPMY-1 cell viability after CYC treatment and calculated the half-maximal inhibitory concentration (IC50) of CYC for the cytotoxic effect. We found that the IC50 for the cytotoxic effect of BPH-1 and WPMY-1 cells was 29.8 and 32.6 μM, respectively (Fig. 3A). Based on the IC50, 5, 10, and 20 μM dosages of CYC were chosen. As shown in Fig. 3B, C, SMO and GLI1–3 were dose-dependently downregulated at the transcriptional and translational level in BPH-1 cells. For GLI2 and GLI3, it was more obviously inhibited in WPMY-1 cells, especially at a high dosage of CYC. Moreover, the CCK-8 assay showed that CYC dose-dependently inhibited BPH-1 and WPMY-1 cell viability with a significant difference observed at 48 and 72 h (Fig. 3D). Meanwhile, cell fluorescence staining revealed that Ki-67-positive cells per field decreased significantly at 10 and 20 μM (Fig. 3E, F). In addition, the flow cytometry analysis showed a significant increase in the percentage of apoptotic human epithelial cells by 20.24% and 45.81% at 10 and 20 μM, respectively. CYC treatment also resulted in a significant increase of the percentage of apoptotic human stromal cells by 10.72%, 17.34%, and 29.00% at 5, 10, and 20 μM (Fig. 4A, B), respectively. Furthermore, the flow cytometry analysis also showed that 20 μM of CYC led to an increase of the G0/G1 phase cells and a reduction of the G2/M phase cells by 14.58% and 12.73% for BPH-1 cells, while a significant increase of the G0/G1 phase cells and a decrease of the G2/M phase cells by 9.37% and 6.84% at 20 μM for WPMY-1 cells, respectively (Fig. 4C, D). The alterations of proteins associated with cell apoptosis and cell cycle were further analyzed and the upregulation of the apoptosis inducer BAX and downregulation of a suppressor of apoptosis Bcl-2 in the CYC-treated BPH-1 and WPMY-1 cells were detected. Meanwhile, caspase-3, a downstream protein of Bcl-2 and BAX in the apoptotic cascade, was decreased, with its active form cleaved caspase-3 increased. Cyclin D1, CDK2, and CDK4 were all decreased in the CYC-treated BPH-1 and WPMY-1 cells, with more obviously at 20 μM of CYC (Fig. 4E).

### CYC treatment induced a reduction of fibrosis markers in WPMY-1 cells

**Fig. 5:**
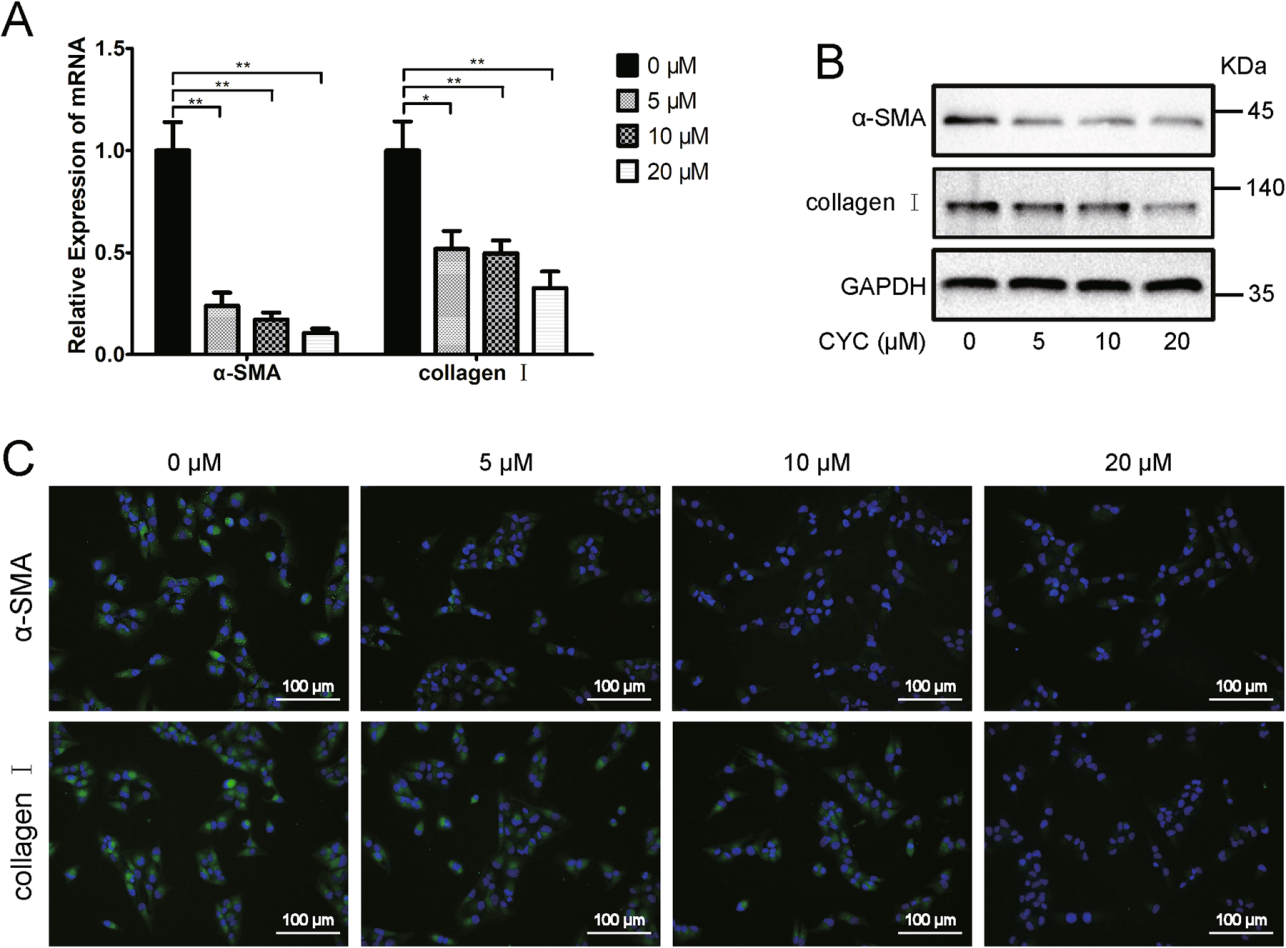
Downregulation of SMO reduced fibrosis markers. **A** The relative mRNA level of fibrosis markers α-SMA and collagen I after CYC treated in WPMY-1. **B** The relative protein level of fibrosis markers α-SMA and collagen I after CYC treated in WPMY-1. **C** Immunofluorescence staining of α-SMA (green) and collagen I (green) in WPMY-1 after CYC treated. *p < 0.05, **p < 0.01; one-way ANOVA. The scale bars are 100 μm.

As shown in Fig. 5 A, B, the fibrosis markers α-smooth muscle actin (α-SMA) and collagen I were downregulatedat both mRNA and protein levels in the CYC-treated WPMY-1 cells. Similarly, the fluorescence intensity of α-SMA and collagen I was decreased in the CYC-treated WPMY-1 cells (Fig. 5 C).

### CYC may suppress BPH via decreasing SMO and GLI1–3 in vivo

**Fig. 6:**
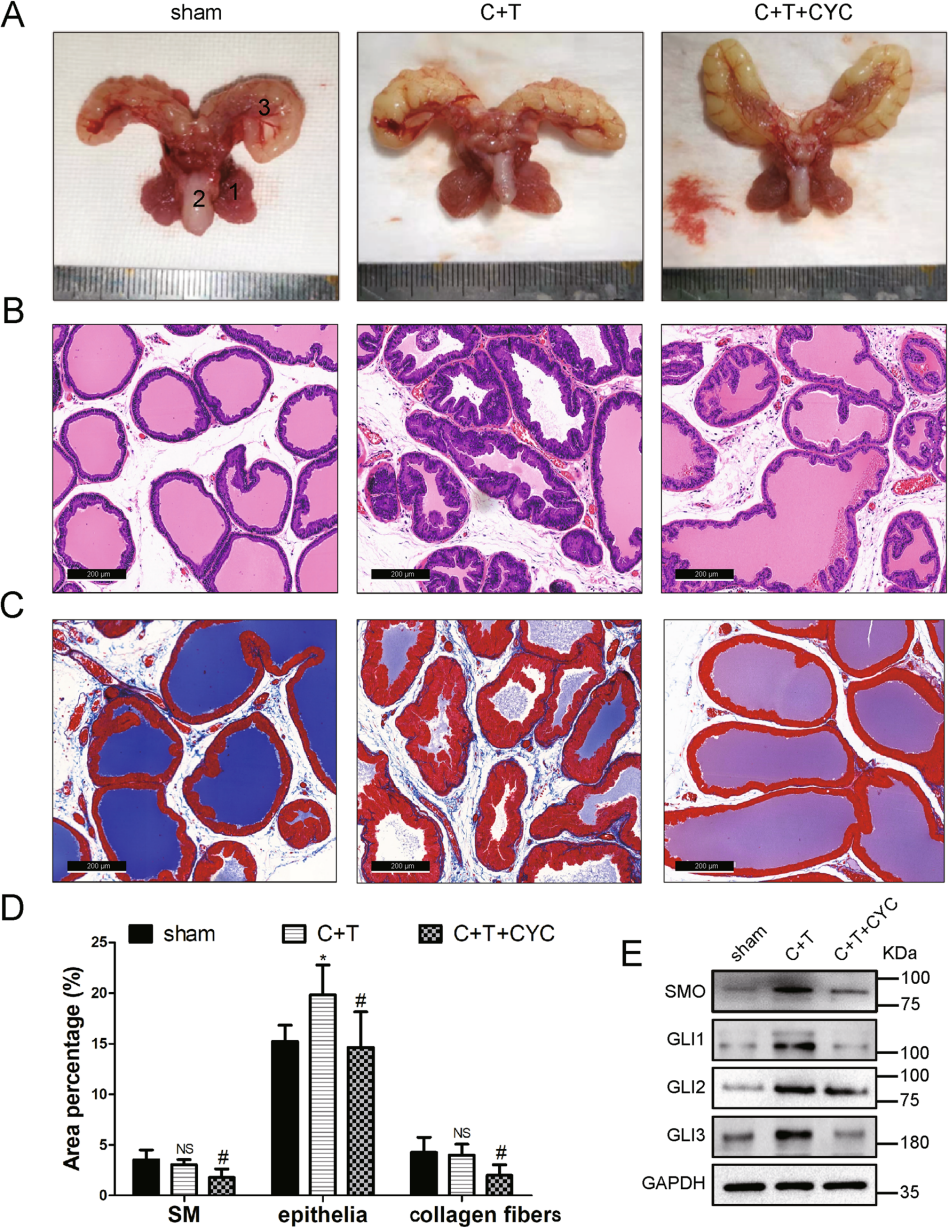
CYC may suppress BPH via decreasing SMO and GLI family in vivo. **A** The rat urogenital tissues from sham, BPH, and CYC-treated rats. (1) ventral prostate, (2) seminal vesicle, and (3) bladder. **B** Representative H&E staining of sham, BPH, and CYC-treated rat prostates. **C** Masson’s trichrome staining of sham, BPH, and CYC-treated rat prostates; prostate epithelial cells were stained orange, SM cells were stained red, and collagen fibers were stained blue. **D** Quantification of Masson’s trichrome staining. **E** Immunoblot assay of SMO and GLI family in sham, BPH, and CYC-treated rats. *n* = 12 for each group. NS means no significance, *p < 0.05 BPH vs Control, ^#^*p* < 0.05 CYC vs BPH; one-way ANOVA. The scale bars are 200 μm.

The in vivo effect of CYC treatment was investigated in rat ventral prostates. The weight of both androgen-sensitive organs (the ventral prostate and seminal vesicles) was obviously increased for castrated rats with 4-week T supplementation (BPH model) (Fig. 6A and Supplementary Table S5). Interestingly, the BPH rats had a significant reduction in body weight during this experimental period (Supplementary Table S5, *p* < 0.01), which may be due to the physiological effect of T. In addition, the ventral prostate weight and prostate index [prostate wet weight (mg)/body weight (g)] were decreased by 22% and 13% in CYC-treated rats (Fig. 6A and Supplementary Table S5), respectively (*p* < 0.05). However, the increased weight of the body and seminal vesicles in CYC-treated rats did not reach statistical significance. Histologically, in BPH rats, the epithelium component was relatively increased, large glands lined with tall columnar epithelium, which are stratified, pseudostratified, or papillary fronds protruding into the lumen. Nevertheless, SMO blockade by CYC effectively prevented the progression of BPH induced by castration with TP supplementation. The shrunk glands were lined with a single layer of columnar epithelium to low cuboidal cells, along with slight edema (Fig. 6B). Masson’s trichrome staining further showed that the hyperplasia of the prostate occurred mainly in the epithelium (increased by 1.3-fold, *p* < 0.05, whereas the stromal component had no difference (either collagen fibers or SM)) in BPH rats. Interestingly, the stromal component was relatively decreased (a 41% loss of SM and a 50% loss of collagen fibers) and the epithelium component was also reduced by 26% in the CYC-treated BPH rat model (Fig. 6C, D). In addition, CYC treatment downregulated the protein level of SMO and GLI1–3 (Fig. 6E).

### CYC induced apoptosis and reduced tissue fibrosis markers in vivo

**Fig. 7:**
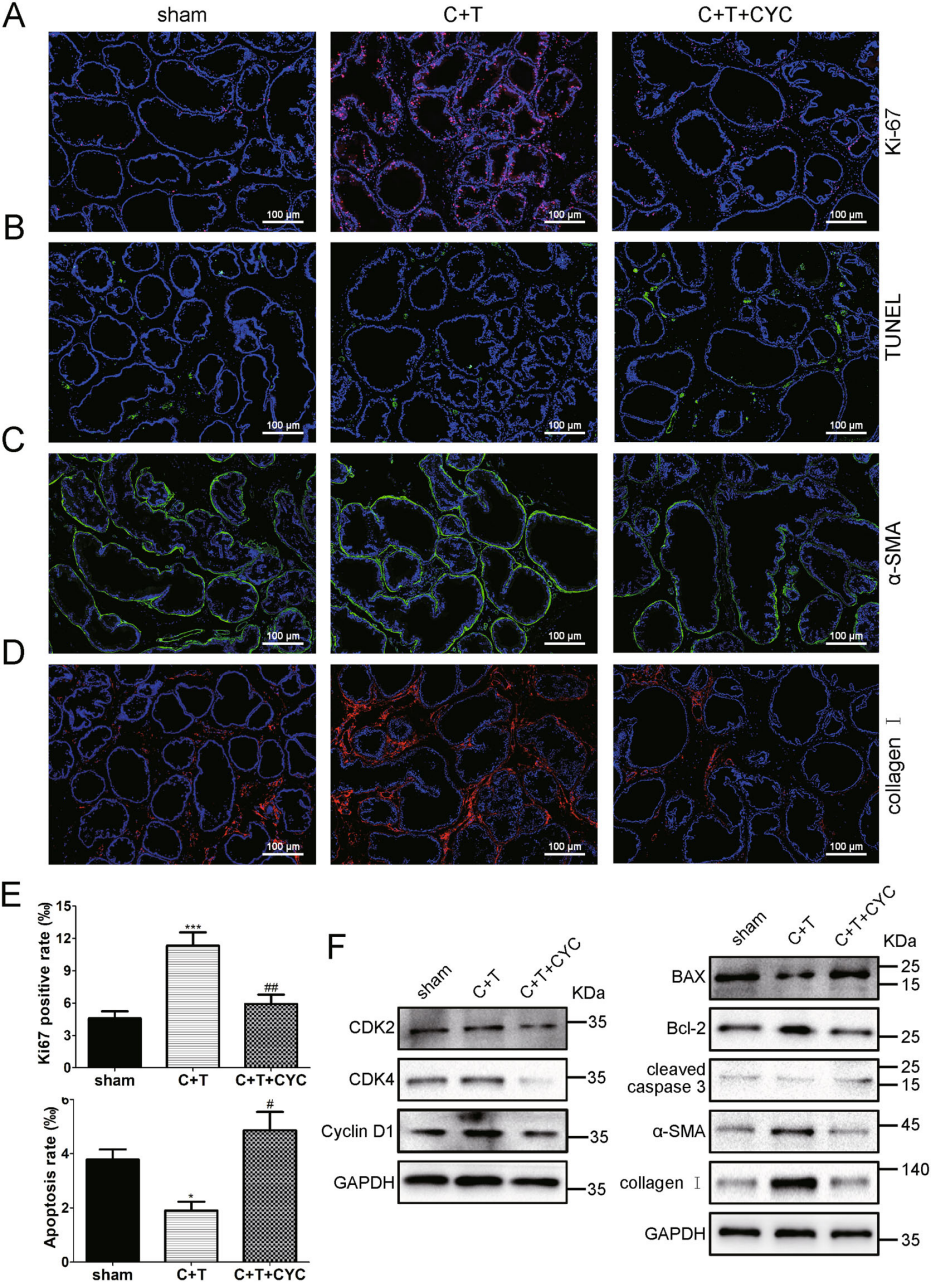
CYC induced apoptosis and reduced tissue fibrosis markers in vivo. **A** The Ki-67 staining for the prostates from sham, BPH, and CYC-treated rats, respectively. DAPI (blue) and fluorescence-labeled images (red) are merged. **B** The TUNEL staining for the prostates from sham, BPH, and CYC-treated rats, respectively. DAPI (blue) and fluorescence-labeled images (green) are merged. **C**, **D** Representative the immunofluorescence staining of α-SMA and collagen I in prostate tissues from sham, BPH and CYC-treated rats. DAPI (blue) indicates nuclear staining, Cy3 immunofluorescence (red) indicates collagen I protein, Cy3-immunofluorescence (green) indicates the α-SMA protein, and merged images were shown. **E** The bar graph for Ki-67-positive rate (%) and apoptosis rate (%) of TUNEL-positive cells. **F** Immunoblot assay of cyclin D1, CDK2, CDK4, BAX, Bcl-2, cleaved caspase-3, α-SMA, and collagen I in sham, BPH, and CYC-treated rats. *p < 0.05 BPH vs sham, ***p < 0.001. BPH vs sham. ^#^*p* < 0.05 CYC vs BPH, ^##^*p* < 0.01 CYC vs BPH. One-way ANOVA. The scale bars are 100 μm.

The BPH rats had a nearly 2.5-fold increase in Ki-67-positive rate (Fig. 7A, E) and a 50% decrease in apoptosis rate detected via the terminal deoxynucleotidyl transferase dUTP nick-end labeling (TUNEL) assay (Fig. 7B, E) in rat prostate. Consistent with previous cell experiments, an increase of apoptosis rate (2.6-fold) and a decrease of Ki-67-positive rate (45% loss) was implied after CYC treatment. Meanwhile, the alterations of proteins associated with apoptosis and cell cycle were measured by western blotting, exhibiting upregulation of BAX and downregulation of Bcl-2, cleaved caspase-3, CDK2, CDK4, and cyclin D1 in CYC-treated rats (Fig. 7F). Furthermore, the tissue fibrosis markers α-SMA and collagen I were analyzed in rat prostate using western blotting and immunostaining. Consistently, in the CYC-treated rats, both α-SMA and collagen I were downregulated compared with BPH rats (Fig. 7C, D, F).

### Overview of SMO/GLI signaling cascades in BPH

**Fig. 8:**
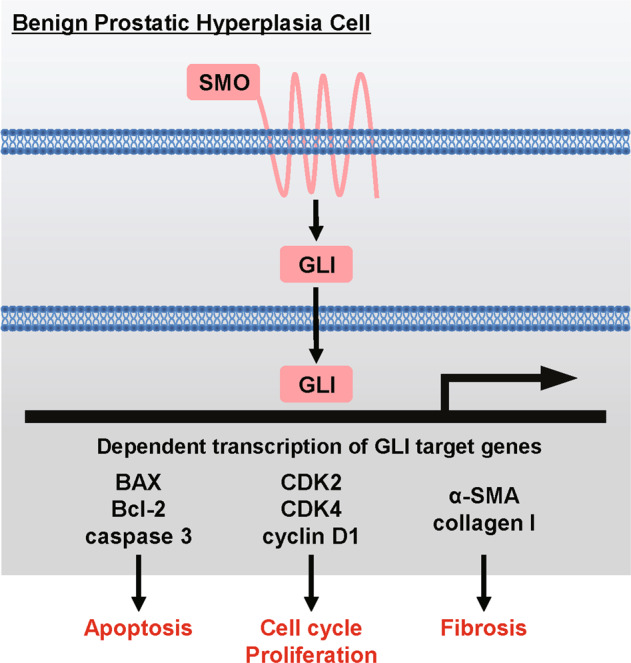
Overview of SMO/GLI signaling cascades in benign prostatic hyperplasia. Canonical SMO/GLI signaling regulates the transcription of target genes, such as *Bcl-2*, *BAX*, *caspase-3*, *CDK2/4*, *cyclin D1*, *α-SMA*, and *collagen I*, which subsequently modulates apoptosis, cell proliferation, cell cycle, and fibrosis through the GLI-dependent transcription network in benign prostatic hyperplasia.

As shown in Fig. 8, canonical SMO/GLI signaling regulates the transcription of target genes, such as *Bcl-2*, *BAX*, *caspase-3*, *CDK2/4*, *cyclin D1*, *α-SMA*, and *collagen I*, which subsequently modulates apoptosis, cell proliferation, cell cycle, and fibrosis through the GLI-dependent transcription network in BPH^12^.

### GLI2 and GLI3 were correlated with some clinical parameters of BPH patients

Finally, we calculated the percentage of the positive area of these proteins on tissue microarray (TMA) of 104 BPH patients and analyzed the correlation between the expression of these proteins and clinical parameters. As shown in Supplementary Figs. S2 and S3, SMO and GLI1–3 stained positive in both the stromal and the epithelium compartments of the human prostate. Interestingly, GLI2 was correlated positively with nocturia and negatively with free prostate-specific antigen (fPSA) (Supplementary Table S6). GLI3 was in a positive relationship with International Prostate Symptom Score (IPSS) and nocturia (Supplementary Table S6). However, we did not find any significant correlation between SMO or GLI1 with any clinical parameter (Supplementary Table S6).

## Discussion

Our novel data demonstrated that SMO and GLI1–3 were localized in the stroma and the epithelium compartments of human prostate tissues and upregulated in hyperplastic prostate tissues. We also showed that SMO cascade inhibition could induce cell apoptosis, cell cycle arrest at the G0/G1 phase, and a reduction of tissue fibrosis markers both in vitro and in vivo. Our study suggested that SMO system could play a vital role in the development of BPH.

An analysis of the human tissue-specific expression by genome-wide integration of transcriptomics^15^ showed that SMO and GLI1–3 were broadly expressed in the human ovary, endometrium, and other tissues, including prostate. In our current study, SMO cascade was abundantly expressed in human prostate tissues, rat ventral prostates, and cultured prostate cell lines. There are adequate experimental evidences demonstrating that prostatic stromal and epithelial cells maintain a sophisticated autocrine/paracrine type of communication^10,16,17^. Therefore, it is necessary for our subsequent study to determine that SMO cascade proteins can either be expressed solely in the epithelia or in both epithelia and stroma. Zhu et al.^18^ have reported that SMO was mainly detected in the ductal epithelia with minimal to moderate expression in the stromal cells of human fetal prostates. A previous TMA analysis^19^ also showed that SMO cascade was localized in both the stromal and the epithelium compartments of adult prostate tissues, which was consistent with our current observation. The high expression of SMO cascade indicated that it could play a role in established BPH by modulating this autocrine/paracrine signaling pathway. Indeed, SMO, GLI2, and GLI3 were all significantly upregulated in BPH when compared with normal ones analyzed through two public databases (GEO and Oncomine). However, GLI1 was found to be significantly increased in BPH in our current study, which may attribute to a limited sample size of BPH or NP accessed by a public database. Besides, the sample size (eight BPH samples and eight control samples) in our current study was much larger than those public datasets of BPH. The altered expression of SMO cascade was further demonstrated via RT-PCR and western blotting. The expression levels of SMO cascade were upregulated in the human hyperplastic prostate and BPH rat prostate when compared with normal controls.

To explore the potential functional activities of SMO cascade involved in the progression of BPH, two common human prostate cell lines BPH-1 and WPMY-1 were selected. SMO downregulated prostate cell model was generated using CYC treatment. CYC, a natural plant-derived alkaloid, acts by inhibiting cellular response to SMO cascade^20,21^. We observed that CYC could induce delayed cell proliferation, cycle arrest at the G0/G1 phase, and apoptosis for prostate cells. The percentage of apoptotic epithelial cells (45.81%) was higher than that in stromal cells (29.00%) at 20 μM of CYC, which could be explained by the fact that prostatic epithelial cells might be more sensitive to a high dosage of CYC than stromal cells in vitro. Proteins involved in apoptosis were strongly altered, especially a high BAX/Bcl-2 ratio, which is involved in mitochondrial-dependent apoptosis^22^, and induce a subsequent activation of caspase- 3 and -7 down in the intrinsic apoptotic pathway^23,24^. Also, it was found that proteins related to cell cycle regulation, such as cyclin D1 and CDK2/4, were all reduced after CYC treatment.

Interestingly, CYC incubation lowered the mRNA and protein levels of fibrosis markers α-SMA and collagen I in cultured immortalized prostate stromal cells WPMY-1. Fibrosis, as a hallmark of pathologic remodeling in many tissues, has adverse effects in many organs and leads to numerous clinical diseases^25–28^. During the past decades, several studies have demonstrated that fibrosis may act as a risk factor contributing to BPH/LUTS etiology^29–32^. Mechanistically, in response to exposure to the canonical profibrotic protein TGF-β1 and other inflammatory proteins^33^, prostate stromal fibroblasts seem to be able to differentiate into myofibroblasts and can be triggered to express fibrosis-associated collagen 1 and 3 and α-SMA^34^. As CYC has a cytotoxic effect, we calculated the IC50 of CYC for the cytotoxic effect of BPH-1 and WPMY-1 cells (29.8 and 32.6 μM, respectively). Therefore, in this current study, we chose 5, 10, and 20 μM dosages for human prostate cell lines and 10 mg/kg of CYC for in vivo intraperitoneal (i.p.) injection. Thus, CYC induced apoptosis, delayed cell growth, and reduced fibrosis markers in the prostate independent of SMO inhibition, rather than an off-target effect or a cytotoxic effect.

Over the past few decades, various types of animal models of BPH have been established to provide useful tools for the study of BPH, including spontaneous BPH models, T-induced BPH models, xenograft models, etc.^35^. However, there is no animal model that could totally mimic all characteristics of human BPH. In our current study, BPH was induced in rats by castration with TP supplementation, which was a widely used, convenient, time-saving, and inexpensive model. Moreover, our TP-induced BPH model was verified with the significantly increased weight of the androgen-sensitive organs (prostate and seminal vesicle) when compared with sham rats. In line with previous observations^36–38^, T injection mainly led to a notable involution of acinar epithelium hyperplasia, such as increased acinus amount, papillary fronds protruding into the glandular cavities, and thickening of the epithelial layer with no change in the percentage of the stromal component. Interestingly, it was noticed that the loss of body weight in BPH rats seems to be due to the exogenous T supplementation inducing an improvement of metabolic characteristics and probably exerts its effects by a direct action on lean body mass. Meanwhile, in the CYC-treated group, BPH rats were treated with 10 mg/kg CYC (i.p.) once a day for 14 days by observing the reduction of ventral prostate weight and shrunk glands lined with a single layer of columnar epithelium to low cuboidal cells. Furthermore, both the stromal and the epithelium components were significantly reduced in the CYC-treated BPH rat model compared with BPH rats. In addition, SMO cascade proteins were upregulated in BPH rat prostate and downregulated with CYC administration. The Ki-67-positive cell rates were attenuated, whereas apoptosis rates were enhanced. The alteration of proteins involved in apoptosis and cell cycle were detected by western blotting by observing an increasing expression of BAX and a decreased expression of CDK2/4, cyclin D1, bcl-2, and cleaved caspase-3. Moreover, downregulation of SMO resulted in a suppressed status for the fibrosis markers α-SMA and collagen I in vivo. All these changes are in parallel with our in vitro findings.

Recently, Yuan and colleagues had reported that CYC administration suppressed BPH by inhibiting epithelial and stromal cell proliferation^39^. In their study, only primary cultured rat prostate cells and rat models were used. No human data were available. Moreover, SMO was not determined in their study. Importantly, they lacked a more exact and scientific process of creating an age-related BPH rat model. Their BPH rats are only 6–8 weeks old, which is hard to find in enlarged prostate.

Finally, a TMA of 104 BPH tissues was constructed to analyze the correlation between the expression of SMO cascade proteins and corresponding clinical parameters. The GLI2 was correlated positively with nocturia and negatively with fPSA and the GLI3 was in a positive relationship with IPSS score and nocturia via a Pearson correlation analysis. The IPSS is recommended as the symptom scoring instrument that is used for the baseline assessment of symptom severity in men with LUTS^40^, which includes the assessment of nocturia. Nocturia, common among older individuals with insomnia, affects people of different ages, races, and genders all over the world. The prevalence of nocturia in both men and women increased with age^41^. In fact, men with BPH more commonly present with irritative than obstructive symptoms, and the most common presenting symptom is nocturia. Our findings suggested that SMO cascade, especially GLI2 and GLI3, was in positive correlation with the progression of BPH. However, the correlation of the SMO system with clinical data is worth further investigation.

Collectively, our present study demonstrated the expression and functional activities of SMO cascade in the prostate. SMO cascade is localized in both the stroma and the epithelium compartments of human prostate tissues and it is upregulated in hyperplastic prostate tissues. Moreover, SMO cascade appears to be involved in the development of BPH via modulation of imbalance of cell apoptosis, cell proliferation, and cell cycle progression, as well as the fibrosis process in vivo and in vitro. Besides, GLI2 and GLI3 are positively correlated with the progression of BPH. Thus, our data suggest that SMO cascade may represent a potential therapeutic target for the treatment of BPH in the future.

## Materials and methods

### Public data acquiring

Three gene expression profiling datasets (GSE6099^14^, GSE3868^42^, and GSE119195^43^) were obtained from GEO (https://www.ncbi.nlm.nih.gov/geo/) of the National Center for Biotechnology Information and the mRNA expression profiles of SMO cascades between human BPH specimens and human NP specimens were analyzed. Meanwhile, the Oncomine database (https://www.oncomine.org/) was used for the transcriptional validation of our concerned genes.

### Animals and tissues

The design of this experiment is outlined in Supplementary Fig. S1. A total of 36 specific pathogen-free grade male Sprague–Dawley rats (6 weeks old) weighing 200–250 g were used and randomly divided into three groups (*n* = 12 per group): sham group, perineal incision + corn oil (MedChemExpress, China) injection (subcutaneously (s.c.)) + dimethyl sulfoxide (DMSO) (MedChemExpress, China) administration (intraperitoneally); C + T group, bilateral orchiectomy + T (T propionate, Sigma-Aldrich, St. Louis, MO) (2 mg/day)/corn oil injection (s.c.) + DMSO administration (i.p.); C + T + CYC group, bilateral orchiectomy + T (2 mg/day)/corn oil injection (s.c.) + CYC (cyclopamine, MedChemExpress, China) administration (10 mg/kg/day, i.p., according to two previous studies^44,45^). Rats were euthanized under anesthesia using a euthanasia solution containing 10% [w/v] chloral hydrate on day 37, and ventral prostates and seminal vesicles were harvested and weighed. The tissues were snap frozen in liquid nitrogen and stored at −80 °C for subsequent molecular analyses or put into 10% neutral-buffered formalin for histological examination. All surgical procedures were performed under anesthesia by i.p. injection of pentobarbital sodium (35 mg/kg; Abbott Laboratory, Chicago, IL). We are blinded to the group allocation when assessing the in vivo effect of CYC on rat prostates. Animal experiments were conducted at the Animal Center of Zhongnan Hospital of Wuhan University and all animal protocols were approved by the Medical Ethics Committee for Experimental animals of Zhongnan Hospital of Wuhan University.

Eight prostate samples from young brain-dead men (mean age, 28.2 ± 4.4 years old) undergoing organ donation were obtained as controls. A total of 104 specimens of BPH samples with clinical data were obtained from the patients who underwent transurethral resection prostate at the Department of Urology, Zhongnan Hospital of Wuhan University. Postoperative prostate pathology examination revealed BPH. Prostate tissues were divided into two strips and were, respectively, stored in liquid nitrogen for PCR and western blotting analysis and stored in 10% neutral-buffered formalin for immunofluorescence microscopy and the construction of TMA. Collection and treatment of all human specimens were carried out in accordance with the approved guidelines of the Ethics Committee at Zhongnan Hospital of Wuhan University.

### The construction and immunohistochemistry analysis of TMA

The summarized clinical data of 104 BPH patients was present in Supplementary Table S1. The paraffin‐embedded specimens were sliced followed by hematoxylin and eosin (H&E) staining. The representative areas of the H&E staining sections were evaluated and confirmed by a senior pathologist. A TMA marker was designed by using 1.5 mm tissue core in each case. Finally, TMA contained 16 × 10 tissue cores for all BPH specimens in each were obtained and then sliced continuously into 4‐μm‐thick sections. Briefly, paraffin sections were deparaffinized first, then antigen retrieval was performed in citrate buffer (pH 6.0), and endogenous peroxidase activity was blocked in 0.3% H_2_O_2_. Subsequently, all slides were incubated with primary and secondary antibodies (listed in Supplementary Tables S2 and S3) until visualization by peroxidase and 3, 3′-diaminobenzidine tetrahydrochloride. All the stained sections were imaged using Olympus-DP72 light microscope (Olympus, Japan). The expression of SMO and GLI1–3 in the prostate tissues from the TMA was blindly quantified by two pathologists. The percentage of protein-positive area was measured by Image J.

### Cell culture

Human benign prostatic enlargement epithelial cell line BPH-1 (Cat. #BNCC339850) was purchased from the Procell Co., Ltd. in Wuhan, China. Identification of the cell lines was performed at the China Center for Type Culture Collection in Wuhan, China. SV40 large-T antigen-immortalized stromal cell line WPMY-1 (Cat. #GNHu36) was purchased from the Stem Cell Bank, Chinese Academy of Sciences in Shanghai, China. The BPH-1 cells were cultured in RPMI-1640 medium (Gibco, China) containing 10% fetal bovine serum (FBS) (Gibco, Australia). The WPMY-1 cells were cultured in Dulbecco’s modified Eagle’s medium (Gibco, China) containing 1% penicillin G sodium/streptomycin sulfate and 5% FBS. All the cell lines were recently authenticated and were cultured in a humidified atmosphere consisting of 95% air and 5% CO_2_ at 37 °C.

### Flow cytometry analysis

For cell cycle analysis, BPH-1 and WPMY-1 cells (1 × 10^6^ cells) were harvested, washed with phosphate-buffered saline (PBS), and then centrifuged. Pellets were resuspended with 1 ml DNA staining solution, which contained 50 μg/ml propidium iodide and 0.1 mg/ml RNaseA, and 10 μl permeabilization solution (Multisciences, China). The DNA content distribution was analyzed by flow cytometry analysis (Beckman, Cat. #FC500, USA) after incubation in the dark at 37 °C for 30 min. For cell apoptosis analysis, fluorescein isothiocyanate (FITC) Annexin V Apoptosis Detection Kit I (BD Biosciences, USA) was used. BPH-1 and WPMY-1 cells (1 × 10^6^ cells) were harvested and then stained with FITC Annexin V Apoptosis Detection Kit I according to the manufacturer’s instruction.

### CCK-8 assay for cell viability

The viability of cells was examined by CCK-8 (MedChemExpress, China) assay. Briefly, the neurons (~5000 cells/well) were seeded in poly-l-lysine-coated 96-well plates and subjected to various treatments as described above. CCK-8 solution (10 μl/100 μl) was added to each culture well, and neurons were incubated for 2 h at 37 °C. Finally, we measured the absorbance at 450 nm with a microplate reader (Thermo Labsystems, Vantaa, Finland).

### Cell immunofluorescence staining

For cell immunofluorescence microscopy, cells were cultured as aforementioned, followed by seeding on 12 mm coverslips and washing by ice-cold (PBS (pH = 7.4). The coverslips were then fixed with 4% paraformaldehyde (PFA) for 30 min, followed by 0.1% Triton X-100 incubation, and then blocked in goat serum for 30 min at room temperature. Afterward, they were incubated with primary antibody (listed in Supplementary Table S2) at room temperature for 2 h, washed with PBS, and incubated with Cy3- or FITC-labeled secondary antibody (listed in Supplementary Table S3) for 1 h. Nuclei were labeled with DAPI (4′,6-diamidino-2-phenylindole) (2 μg/ml). Visualization was done with a laser scanning confocal microscope (Olympus, Tokyo, Japan).

### RNA isolation, reverse transcription, and quantitative real-time PCR (qRT-PCR) analysis

Total RNA was isolated from frozen tissues and cell lines using Trizol reagent (Invitrogen, Carlsbad, CA, USA) according to the manufacturer’s instructions and quantitated at 260/280 nm using a NanoPhotometer spectrophotometer (IMPLEN, Westlake Village, CA, USA). Two micrograms of total RNA was reverse transcribed to complementary DNA via the SuperScript II First-Strand Synthesis System according to the manufacturer (Invitrogen, Carlsbad, CA, USA). QRT-PCR was performed to determine the level of mRNA expression of a gene of interest based on SYBR Green using a Bio-Rad (Hercules, CA, USA) CFX96 system. The expression levels of genes were normalized to the expression of glyceraldehyde 3-phosphate dehydrogenase (GAPDH) mRNA and compared by 2^−ΔΔCT^ method. Primer sequences are listed in Supplementary Table S4. Values were normalized for amplified GAPDH alleles.

### Western blotting analysis

Tissues and cell lines were lysed and ultrasonicated in RIPA reagent containing protease inhibitor and phosphatase inhibitor (Sigma-Aldrich, St. Louis, MO, USA) on ice for 30 min. The supernatant was collected after centrifugation at 14,000 × *g* for 10 min at 4 °C. Then, the protein concentration was measured by bicinchoninic acid assay. Protein extracts were isolated by sodium dodecyl sulfate-polyacrylamide gel electrophoresis and then transferred to a polyvinylidene fluoride membrane (Millipore, Billerica, MA, USA) using a Bio-Rad wet transfer system. The membrane was then blocked in Tris-buffered saline with 0.05% Tween-20 buffer containing 5% skim milk, and incubated sequentially with primary and secondary antibodies (listed in Supplementary Tables S2 and S3). An Enhanced Chemiluminescence Kit (Thermo Fisher Scientific, Waltham, MA, USA) was used to expose the bands.

### H&E staining

Prostate paraffin sections (5 μm) were deparaffinized in xylene for 3 × 10 min, rehydrated in descending concentrations of ethanol (100, 96, 80, and 70%), and H_2_O. The sections were then stained in 10% hematoxylin (Sigma-Aldrich, St. Louis, MO, USA) for 7 min, followed by washing under the tap water for 10 min to reveal the nuclei. Afterward, the sections were stained in 1% eosin (Sigma-Aldrich, St. Louis, MO, USA) containing 0.2% glacial acetic acid for 5 min. After staining, the sections were washed with tap water, dehydrated in increasing grades of ethanol (70, 80, 96, and 100%), and cleared in xylene for 3 × 10 min. The sections were imaged by an Olympus-DP72 light microscope (Olympus, Japan).

### Masson’s trichrome staining

As previously described^46^, prostate tissues were embedded into paraffin after being fixed in 10% formalin for 24–36 h and cut into 5 μm sections. Then, the sections were stained using Masson’s trichrome staining. Staining was detected by light microscopy. Prostatic smooth muscle (SM) cells, collagen fibers, and epithelial cells were stained red, blue, and orange, respectively. In each sample, we analyzed three areas randomly under magnification (×200). The area percentage of SM, collagen fibers, and glandular epithelium were quantitated with Image Pro Plus 5.0.

### TUNEL assay

Rat prostate tissues were fixed in 4% PFA, embedded in paraffin, and then digested with proteinase K for 20 min. The sections were then incubated with a fluorometric DNA Fragmentation Detection Kit (PromoCell, Heidelberg, Germany) according to the manufacturer’s instructions. Nuclei were labeled with DAPI. Visualization was done with a Laser Scanning Confocal Microscope (Olympus, Tokyo, Japan). DAPI and fluorescence-labeled images were merged and TUNEL-positive apoptotic cells in the merged images were quantified by the counting of positively stained cells.

### Tissue immunofluorescent staining

Tissues were sectioned in 10-μm-thick slices and thawed, mounted onto glass slides using a cryostat (Leica CM 1850, Wetzlar, Germany), air-dried, and fixed for 10 min in ice-cold acetone. Slides were washed in PBS and incubated for 2 h in a mixture of PBS supplemented with 0.2% Triton X-100 and 0.1% bovine serum albumin, followed by incubation overnight with the primary antibodies (listed in Supplementary Table S2). The secondary antibodies (listed in Supplementary Table S3) employed to visualize the localization of SMO and GLI1–3 were Cy3-conjugated goat anti-rabbit IgG (1:1000). DAPI was used for staining the nucleus. Visualization was done with a Laser Scanning Confocal Microscope (Olympus, Tokyo, Japan).

### Statistical analysis

All analyses were performed at least three times and represented data from three individual experiments. The data values were expressed as the means ± standard deviation (SD). Statistical analysis was performed using either the Student’s *t* test (two groups compared) or one-way analysis of variance and Tukey’s post tests with SPSS 20.0 (multiple means compared). Statistical significance was considered as a *p* value < 0.05.

## Supplementary information

Supplementary table S1

Supplementary table S2

Supplementary table S3

Supplementary table S4

Supplementary table S5

Supplementary table S6

Supplementary figure legends

Supplementary figure S1

Supplementary figure S2

Supplementary figure S3
