# Supplementary material for: Smoothened inhibition leads to decreased cell proliferation and suppressed tissue fibrosis in the development of benign prostatic hyperplasia
Source: Cell Death Discov. 2021 May 18;7:115. doi: 10.1038/s41420-021-00501-4 (PMC8131753; doi:10.1038/s41420-021-00501-4)
Supplement: Supplementary file 1 — Supplementary table S1 [file 41420_2021_501_MOESM1_ESM.doc]

**Supplementary Table S1 Clinical features of 104 patients with benign prostatic hyperplasia.**

|  | **Mean** | **SD** |
| --- | --- | --- |
| Age（years） | 70.1 | 7.5 |
| Body mass index (kg/m^2^） | 22.8 | 2.8 |
| Prostate volume (cm^3^) | 60.8 | 36.7 |
| Total prostate specific antigen (ng/ml) | 7.0 | 5.9 |
| Free prostate specific antigen (ng/ml) | 1.6 | 1.4 |
| Maximum flow rate (ml/s) | 10.0 | 5.9 |
| Residual urine（ml） | 160.7 | 114.5 |
| International prostate symptom score | 21.7 | 7.4 |
| Nocturia (N) | 3.1 | 2.0 |
