# Supplementary material for: Smoothened inhibition leads to decreased cell proliferation and suppressed tissue fibrosis in the development of benign prostatic hyperplasia
Source: Cell Death Discov. 2021 May 18;7:115. doi: 10.1038/s41420-021-00501-4 (PMC8131753; doi:10.1038/s41420-021-00501-4)
Supplement: Supplementary file 2 — Supplementary table S2 [file 41420_2021_501_MOESM2_ESM.doc]

**Supplementary Table S2 List of primary antibodies.**

| **Antigens** | **Name of antibody** | **Supplier** | **Species antibodies raised in** | **Dilution used** |
| --- | --- | --- | --- | --- |
| SMO | SMO antibody | Abclonal  A3274 | Rabbit  Polyclonal antibody | 1:1000 (WB)  1:100 (IF) |
| GLI1 | GLI1 antibody | Abclonal  A14675 | Rabbit  Polyclonal antibody | 1:1000 (WB)  1:100 (IF) |
| GLI2 | GLI2 specific antibody | Proteintech  18989-1-AP | Rabbit  Polyclonal antibody | 1:500 (WB)  1:50 (IF) |
| GLI3 | GLI3 specific antibody | Proteintech  19949-1-AP | Rabbit  Polyclonal antibody | 1:500 (WB)  1:50 (IF) |
| CDK2 | CDK2 (78B2) | Cell Signaling  Technology, #2546 | Rabbit  Monoclonal antibody | 1:1000 (WB) |
| CDK4 | CDK4 (D9G3E) | Cell Signaling  Technology, #12790 | Rabbit  Monoclonal antibody | 1:1000 (WB) |
| Cyclin D1 | Cyclin D1 (92G2) | Cell Signaling  Technology, #2978 | Rabbit  Monoclonal antibody | 1:1000 (WB) |
| BAX | BAX antibody | Abclonal  A12009 | Rabbit  Polyclonal antibody | 1:1000 (WB) |
| Bcl-2 | Bcl-2 antibody | Abclonal  A11025 | Rabbit  Polyclonal antibody | 1:1000 (WB) |
| caspase 3 | caspase 3 antibody | Abclonal  A0124 | Rabbit  Polyclonal antibody | 1:1000 (WB) |
| cleaved caspase 3 | cleaved caspase 3 (Asp175) (5A1E) | Cell Signaling  Technology, #9664 | Rabbit  Monoclonal antibody | 1:1000 (WB) |
| α-SMA | Anti -alpha smooth muscle Actin antibody | Servicebio  GB13044 | Mouse  Monoclonal antibody | 1:1000 (WB)  1:500 (IF) |
| collagen I | COL1A1 antibody | Abclonal  A1352 | Rabbit  Polyclonal antibody | 1:1000 (WB)  1:200 (IF) |
| Ki-67 | Ki-67 antibody | Servicebio  GB111141 | Rabbit  Polyclonal antibody | 1:1200 (IF) |
| GAPDH | GAPDH antibody | Abclonal  AC027 | Rabbit  Polyclonal antibody | 1:10000 (WB) |
