# Supplementary material for: Smoothened inhibition leads to decreased cell proliferation and suppressed tissue fibrosis in the development of benign prostatic hyperplasia
Source: Cell Death Discov. 2021 May 18;7:115. doi: 10.1038/s41420-021-00501-4 (PMC8131753; doi:10.1038/s41420-021-00501-4)
Supplement: Supplementary file 3 — Supplementary table S3 [file 41420_2021_501_MOESM3_ESM.doc]

**Supplementary Table S3 List of secondary antibodies.**

| **Secondary Detection System Used** | **Host** | **Dilution used** | **Supplier** |
| --- | --- | --- | --- |
| Anti-Mouse-IgG (H + L)-HRP | Goat | 1:10,000 (WB) | Sungene Biotech, Tianjin, China, Cat. #LK2003 |
| Anti-Rabbit-IgG (H + L)-HRP | Goat | 1:10,000 (WB) | Sungene Biotech, Cat. #LK2001 |
| Anti-rabbit IgG (H+L), F(ab')2 fragment (Alexa Fluor® 488 Conjugate) | Goat | 1:50 (IF) | Cell Signaling Technology, USA, cat. no. 4412 |
| Anti-mouse IgG (H+L), F(ab')2 Fragment (Alexa Fluor® 488 Conjugate) | Goat | 1:50 (IF) | Cell Signaling Technology, USA, cat. no. 4408 |
| Hoechst 33342 (1 mg/ml) nucleic acid staining (DAPI) | - | 1:750 (IF) | Molecular Probes/Invitrogen, Carlsbad, CA, USA, cat. no. A11007 |
