# Supplementary material for: Smoothened inhibition leads to decreased cell proliferation and suppressed tissue fibrosis in the development of benign prostatic hyperplasia
Source: Cell Death Discov. 2021 May 18;7:115. doi: 10.1038/s41420-021-00501-4 (PMC8131753; doi:10.1038/s41420-021-00501-4)
Supplement: Supplementary file 4 — Supplementary table S4 [file 41420_2021_501_MOESM4_ESM.doc]

**Supplementary Table S4 Primer sequence used for qRT-PCR.**

| Target genes | | Human（5’ to 3’） |
| --- | --- | --- |
| SMO | Forward | ATCTCCACAGGAGAGACTGGTTCGG |
|  | Reverse | AAAGTGGGCCTTGGGAACATG |
| GLI1 | Forward | TACTCACGCCTCGAAACCT |
|  | Reverse | GTCTGCTTTCCTCCCTGATG |
| GLI2 | Forward | GCCCTTCCTGAAAAGAAGAC |
|  | Reverse | CATTGGAGAAACAGGATTGG |
| GLI3 | Forward | GAAGTGCTCCACTCGAACAGA |
|  | Reverse | GTGGCTGCATAGTGATTGCG |
| α-SMA | Forward | GGCATTCACGAGACCACCTAC |
|  | Reverse | CGACATGACGTTGTTGGCATAC |
| collagen I | Forward | GAGGGCCAAGACGAAGACATC |
|  | Reverse | CAGATCACGTCATCGCACAAC |
| GAPDH | Forward | ATCCCATCACCATCTTCCAGGAG |
|  | Reverse | CCTGCTTCACCACCTTCTTGATG |
