# Supplementary material for: Smoothened inhibition leads to decreased cell proliferation and suppressed tissue fibrosis in the development of benign prostatic hyperplasia
Source: Cell Death Discov. 2021 May 18;7:115. doi: 10.1038/s41420-021-00501-4 (PMC8131753; doi:10.1038/s41420-021-00501-4)
Supplement: Supplementary file 5 — Supplementary table S5 [file 41420_2021_501_MOESM5_ESM.doc]

**Supplementary Table S5 Variation of biometric and physiological parameters in sham, C+T and C+T+CYC rats.**

| Group | Body weight(g) | Ventral prostate  weight(mg) | Seminal vesicles  weight(mg) | Prostate  index |
| --- | --- | --- | --- | --- |
|  | Initial Final |  |  |  |
| sham | 223.0(17.2) 436.4(40.3) | 558.0(62.6) | 1614.0(127.0) | 1.3(0.1) |
| C+T | 230.6(13.2) 378.3(20.2) **^**^** | 668.6(96.0) **^*^** | 2658.6(668.5) **^**^** | 1.8(0.2) **^**^** |
| C+T+CYC | 231.4(20.2) 387.7(20.4) | 524.3(82.8) **^#^** | 2890.0(253.8) | 1.4(0.2) **^##^** |

C, castration T, testosterone CYC, cyclopamine.

**^*^***p*＜0.05 C+T vs sham, **^**^***p*＜0.01 C+T vs sham, **^#^***p*＜0.05 C+T vs C+T+CYC, **^##^***p*＜0.01 C+T vs C+T+CYC.
