# Supplementary material for: Smoothened inhibition leads to decreased cell proliferation and suppressed tissue fibrosis in the development of benign prostatic hyperplasia
Source: Cell Death Discov. 2021 May 18;7:115. doi: 10.1038/s41420-021-00501-4 (PMC8131753; doi:10.1038/s41420-021-00501-4)
Supplement: Supplementary file 6 — Supplementary table S6 [file 41420_2021_501_MOESM6_ESM.doc]

| **Supplementary table S6 The correlation analysis of clinical parameters and SMO cascade in 104 BPH patients.** | | | | | | | | |
| --- | --- | --- | --- | --- | --- | --- | --- | --- |
|  | SMO | | GLI1 | | GLI2 | | GLI3 | |
|  | Pearson correlation | *p*-value | Pearson correlation | *p*-value | Pearson correlation | *p*-value | Pearson correlation | *p*-value |
| Age | .068 | .491 | .059 | .549 | .029 | .744 | -.192 | .051 |
| BMI | .057 | .564 | -.076 | .444 | .021 | .837 | .131 | .188 |
| PV | .119 | .243 | .079 | .438 | .023 | .820 | -.021 | .838 |
| IPSS | .068 | .494 | -.060 | .547 | .164 | .098 | ***.325^**^*** | ***.001*** |
| N | .107 | .283 | -.026 | .797 | ***.207^*^*** | ***.036*** | ***.248^*^*** | ***.012*** |
| tPSA | -.128 | .218 | .029 | .783 | -.181 | .081 | -.071 | .496 |
| fPSA | -.141 | .174 | -.112 | .282 | ***-.233^*^*** | ***.024*** | -.058 | .576 |
| Qmax | .169 | .312 | .232 | .161 | .073 | .664 | .196 | .238 |
| RU | -.022 | .902 | -.039 | .823 | -.008 | .966 | .032 | .854 |

| BMI, body mass index. PV, prostate volume. IPSS, international prostate symptom score.  N, Nocturia. tPSA, total prostate specific antigen. fPSA, free prostate specific antigen.  Qmax, maximum flow rate. RU, residual urine. ^*^. *p*<0.05 (2-tailed) ^**^. *p*<0.01 (2-tailed) |
| --- |
