# Supplementary material for: Smoothened inhibition leads to decreased cell proliferation and suppressed tissue fibrosis in the development of benign prostatic hyperplasia
Source: Cell Death Discov. 2021 May 18;7:115. doi: 10.1038/s41420-021-00501-4 (PMC8131753; doi:10.1038/s41420-021-00501-4)
Supplement: Supplementary file 7 — Supplementary figure legends [file 41420_2021_501_MOESM7_ESM.docx]

**Supplementary Figure legends**

**Supplementary Fig. S1 Flow diagram of the experimental procedure.** A rat model of benign prostatic hyperplasia. CYC, cyclopamine; T, testosterone; C, castration.

**Supplementary Fig. S2 Immunohistochemical of SMO and GLI1 on TMA of BPH. A**: SMO **B**: GLI1 The scale bars are 2000μm.

**Supplementary Fig. S3 Immunohistochemical of GLI2 and GLI3 on TMA of BPH. A**: GLI2 **B**: GLI3 The scale bars are 2000μm.
