# Supplementary figures and images for: Smoothened inhibition leads to decreased cell proliferation and suppressed tissue fibrosis in the development of benign prostatic hyperplasia
Source: Cell Death Discov. 2021 May 18;7:115. doi: 10.1038/s41420-021-00501-4 (PMC8131753; doi:10.1038/s41420-021-00501-4)

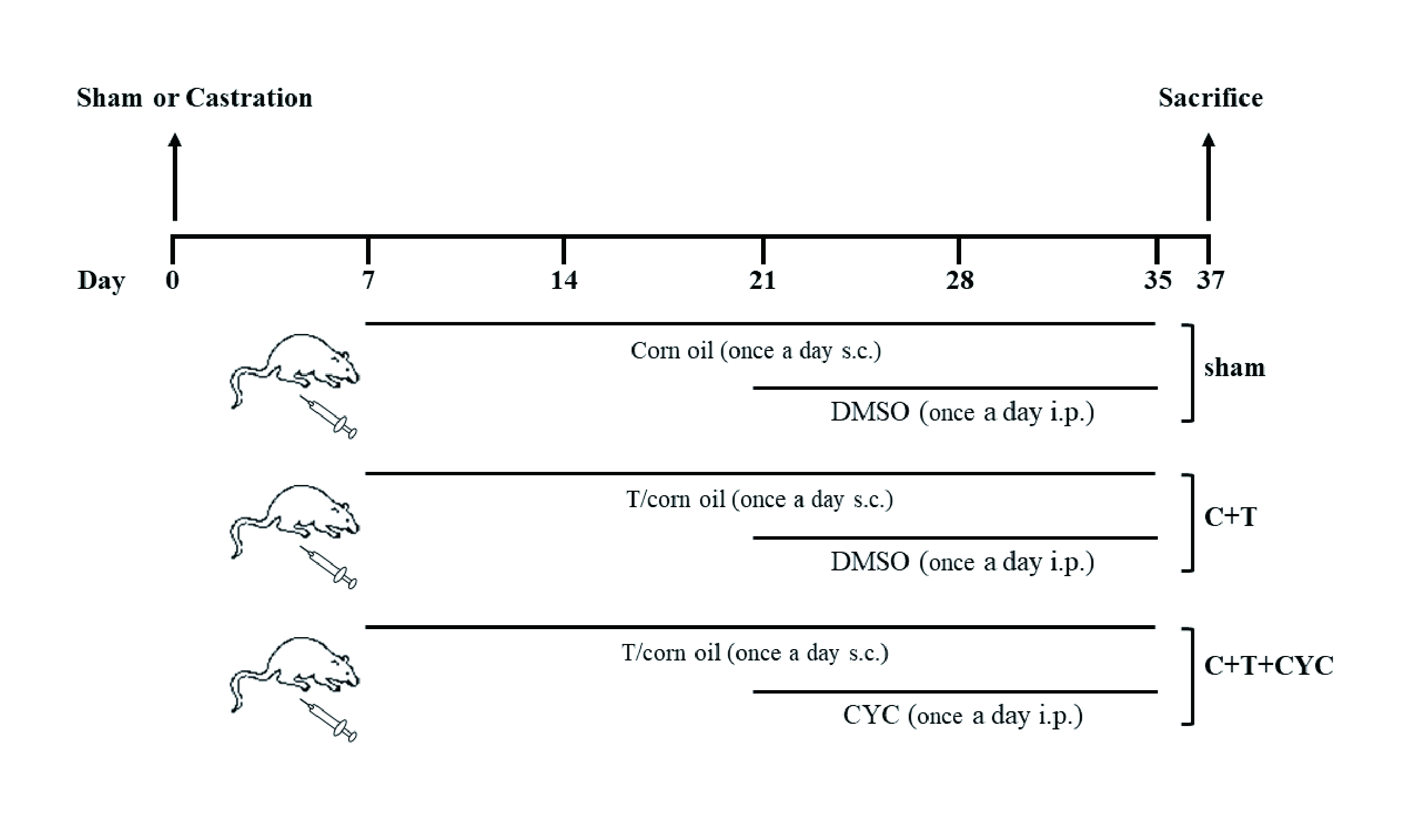

Supplement: Supplementary file 8 — Supplementary figure S1 [file 41420_2021_501_MOESM8_ESM.tif]

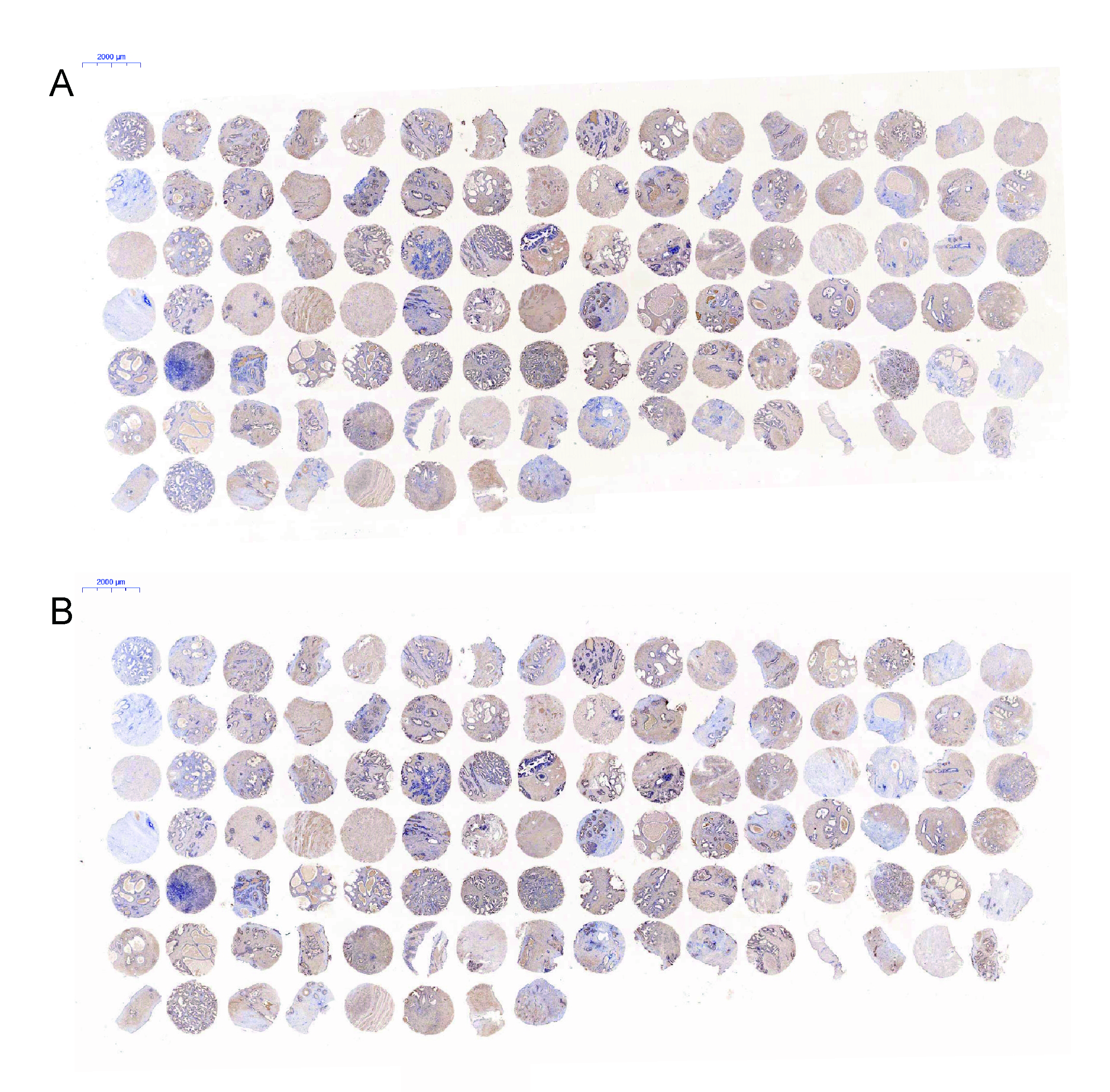

Supplement: Supplementary file 9 — Supplementary figure S2 [file 41420_2021_501_MOESM9_ESM.tif]

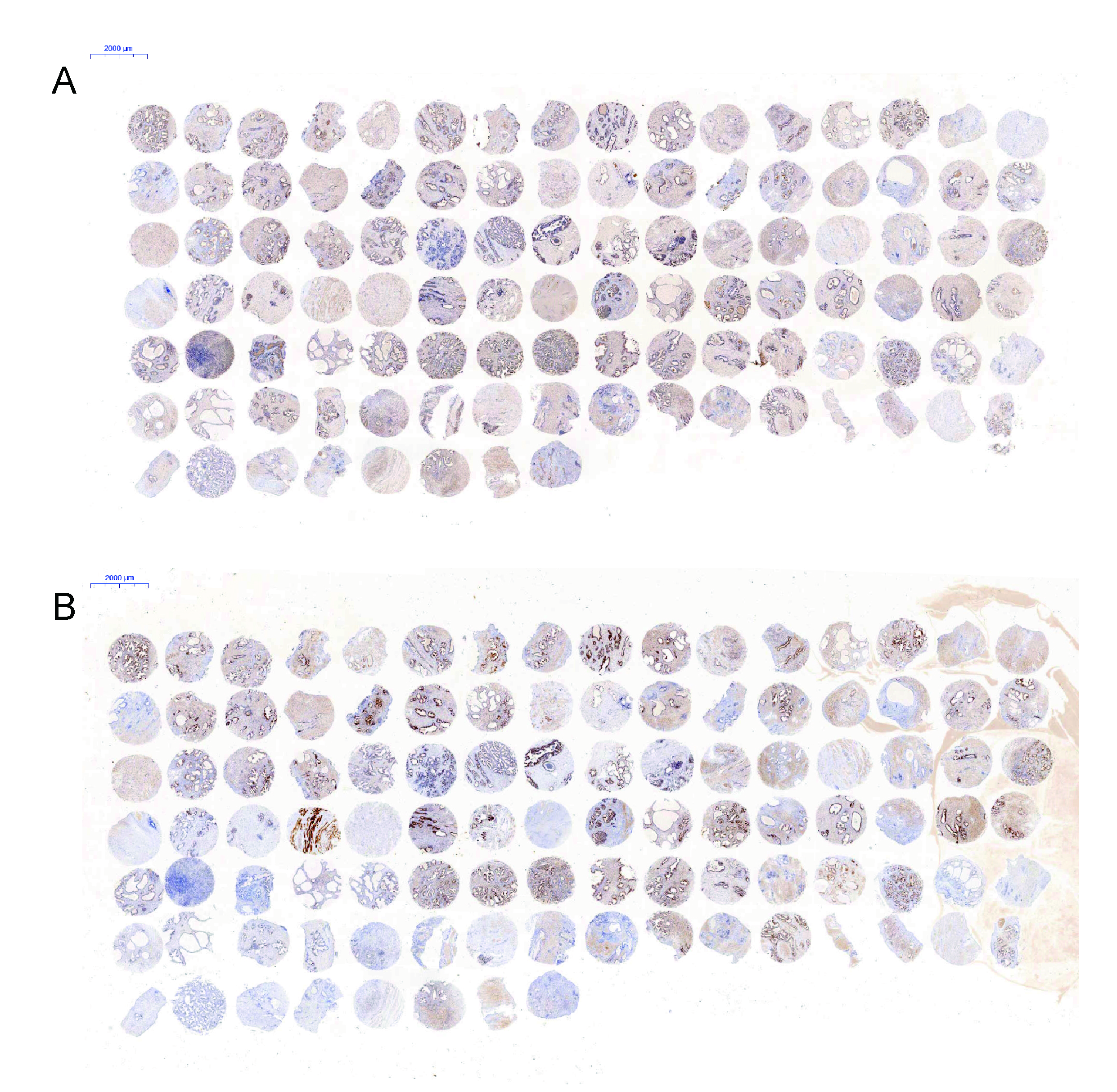

Supplement: Supplementary file 10 — Supplementary figure S3 [file 41420_2021_501_MOESM10_ESM.tif]
